# Physical capability in mid-life and survival over 13 years of follow-up: British birth cohort study

**DOI:** 10.1136/bmj.g2219

**Published:** 2014-04-29

**Authors:** Rachel Cooper, Bjørn Heine Strand, Rebecca Hardy, Kushang V Patel, Diana Kuh

**Affiliations:** 1 MRC Unit for Lifelong Health and Ageing at UCL, London WC1B 5JU, UK; 2 Norwegian Institute of Public Health, N-0473 Oslo, Norway; 3 Department of Anesthesiology and Pain Medicine, University of Washington, Seattle, USA

## Abstract

**Objectives** To examine associations between three commonly used objective measures of physical capability assessed at age 53 and a composite score of these measures and all cause mortality; to investigate whether being unable to perform these tests is associated with mortality.

**Design** Cohort study.

**Setting** MRC National Survey of Health and Development in England, Scotland, and Wales.

**Participants** 1355 men and 1411 women with data on physical capability at age 53 who were linked to the National Health Service (NHS) central register for death notification.

**Main outcome measure** All cause mortality between ages 53 (1999) and 66 (2012).

**Results** For each of the three measures of physical capability (grip strength, chair rise speed, and standing balance time) those participants unable to perform the test and those in the lowest performing fifth were found to have higher mortality rates than those in the highest fifth. Adjustment for baseline covariates partially attenuated associations but in fully adjusted models the main associations remained. For example, the fully adjusted hazard ratio of all cause mortality for the lowest compared with the highest fifth of a composite score of physical capability was 3.68 (95% confidence interval 2.03 to 6.68). Those people who could not perform any of the tests had considerably higher rates of death compared with those people able to perform all three tests (8.40, 4.35 to 16.23). When a series of models including different combinations of the measures were compared by using likelihood ratio tests, all three measures of physical capability were found to improve model fit, and a model including all three measures produced the highest estimate of predictive ability (Harrell’s C index 0.71, 95% confidence interval 0.65 to 0.77). There was some evidence that standing balance time was more strongly associated with mortality than the other two measures.

**Conclusions** Lower levels of physical capability at age 53 and inability to perform capability tests are associated with higher rates of mortality. Even at this relatively young age these measures identify groups of people who are less likely than others to achieve a long and healthy life.

## Introduction

A recent systematic review and additional meta-analyses have provided robust evidence of associations between better performance in objective tests of physical capability, including stronger grip and faster walking speed, and lower rates of all cause mortality in older populations living in the community.^1^ ^2^ Informed by such evidence, these easily administrable tests have been included in recent studies of candidate biomarkers of ageing and have been found to perform well, as assessed by a range of criteria, when compared with more traditional biomarkers including inflammatory markers, blood pressure, and telomere length.^3^ ^4^

Various explanations of the associations between physical capability and mortality have been proposed. Levels of physical capability could reflect both clinically manifest and undetected disease and ageing processes. They could also provide a useful indication of lifetime exposure to risk factors for premature mortality and associated factors (such as physical inactivity, smoking, and low levels of cardiorespiratory fitness).^1^ ^2^ As levels of physical capability in later life are determined not only by the rate of decline generally observed from mid-life onwards but also by the peak achieved during growth and development, associations could also be partially explained by initial developmental differences between individuals that influence both physical capability and rates of survival.^1^

The recent systematic review^1^ highlighted several gaps in the existing literature and by examining some of these it might be possible to elucidate further the explanations for the associations between physical capability and mortality. The first important gap identified was a lack of studies that had examined these associations at younger ages; studies of the associations of walking speed, time to rise from a chair and standing balance performance with mortality had been conducted exclusively in populations aged over 60 at the time of assessment of capability, most populations were aged ≥70 and had follow-up of less than 10 years. Although the review included only study populations of people who were living in the community, the possibility that associations could be explained by the high level of disease burden (that is, both clinical and subclinical pathology) that impacts on physical capability and survival rates at older ages could not be ruled out, even when adjustments for health status had been made. Studies of associations between physical capability and mortality in younger populations who have higher mean levels of physical capability^5^ and less disease burden are essential to elucidate whether associations exist even before the establishment of strong pathways between disease pathology, physical capability, and mortality in later life.

Secondly, there was a lack of studies able to make fair comparisons of the associations between different measures of physical capability and mortality.^6^ As different objective measures of physical capability depend to some extent on the same underlying physiological systems, it is important to assess the value of conducting more than one different test of physical capability and also of deriving a composite score,^7^ especially as time for assessment is often limited in research and applied settings.

Thirdly, it is often not clear how those people unable to perform the tests have been handled in analyses. Even these relatively simple tests have exclusion criteria, and so an important challenge is how to deal with the increasing proportion of people who are unable to perform these tests with increasing age. Studies that use composite scores of physical capability, such as the short physical performance battery, often include those people who are unable to perform the tests by allocating them the lowest possible score (when a lower score indicates worse performance)^8^ but in analyses of performance in individual tests, participants without a valid result are often excluded. This is despite the fact that if data are missing because participants were unable to complete the test for health reasons this might be informative.

To deal with these research gaps, we used data from the MRC National Survey of Health and Development, the oldest British birth cohort study. We used comparable models to examine the associations between three objective measures of physical capability (grip strength, chair rise speed, and standing balance time) assessed at age 53, and a composite score of these measures, and all cause mortality rates over 13 years of follow-up; assessed the added value of each of the measures of physical capability; and examined whether being unable to perform each of these tests was associated with mortality. We also tested whether the main associations were independent of important covariates.

We hypothesised that there would be associations between lower levels of physical capability and higher rates of all cause mortality but that these associations might be non-linear, with stronger associations found among those with lower performance levels. We also hypothesised that all three measures would provide added value as we expected that the correlations between the three different measures of physical capability would be lower at age 53 than in older study populations because of the presence at older ages of more shared risk factors for poor performance.

## Methods

The Medical Research Council National Survey of Health and Development (NSHD) is a socially stratified sample of 5362 singleton births (2547 males and 2815 females) that took place in one week of March 1946 in England, Scotland and Wales, with regular follow-up across life.^9^ In 1999, when study participants were aged 53, 3035 participants (62% of those alive) were successfully contacted, of whom 2984 (98%) received a home visit from a trained nurse. Of the 2327 participants not contacted in 1999, 469 had previously died, 948 had refused to participate, 580 were living abroad, and 330 could not be traced.

### Physical capability at age 53

Physical capability was assessed during home visits at age 53 with three objective measures: grip strength, chair rise time, and standing balance time. Trained nurses conducted these tests using standardised protocols as described elsewhere^10^ and summarised here. These three tests were chosen for assessment at age 53 because they are some of the most commonly used measures of physical capability in epidemiological studies^11^ and were expected to detect meaningful variation in capability between individuals in mid-life across the full spectrum of ability.

Grip strength was measured isometrically with an electronic handgrip dynamometer. The dynamometers were calibrated at the start of testing by using a back-loading rig and are accurate, linear, and stable to within 0.5 kg.^12^ The retest variability within individual participants for maximal voluntary tests of strength in those unused to such measurements is about 9%.^13^ Two values were recorded for each hand and the highest used in analyses. Chair rise time was measured with a stopwatch as the time taken to rise from a sitting to a standing position with straight back and legs and then to sit down again 10 complete times as fast as possible. For high scores to indicate good performance, we calculated chair rise speed by dividing the number of rises (that is, 10) by the time taken to complete 10 rises (in minutes). Standing balance time was measured, using a stopwatch, as the longest time, up to a maximum of 30 seconds, participants could maintain a one-legged stance in a standard position with their eyes closed. For each of the three tests nurses recorded if a study participant was unable or unwilling to perform the test and the reason for this (such as health reasons or technical problems). There is evidence to suggest that all three measures are valid and reliable.^14^ ^15^ ^16^ ^17^ ^18^ ^19^

We created a composite score of physical capability using the method devised by Guralnik and colleagues.^20^ This involved rescaling each of the three test scores on a scale of 0 (low performance) to 1 (high performance) so that all three measures have equal weight in the final score (see appendix for further details of the rescaling procedure); assigning those participants unable to perform each test for health reasons a value of 0; and summing the three rescaled capability scores to create a normally distributed aggregate physical capability score with a range of 0 to 3. We also derived a variable indicating the number of tests participants were unable to perform for health reasons (range 0 to 3).

### Ascertainment of death

All consenting study participants have been linked to the National Health Service (NHS) central register since 1972. For the purposes of these analyses, we included deaths from any cause notified by the register from March 1999 to March 2012.

### Covariates

A priori we identified potential confounders—that is, factors that have previously been shown to be associated with both physical capability and mortality.^10^ ^21^ ^22^ ^23^ ^24^ ^25^ ^26^ ^27^

*Body size*—Height (cm) and weight (kg) were measured by nurses during the home visit when participants were aged 53, and BMI (kg/m^2^) was calculated with these measures. For those people with valid performance data but missing height (n=10), height was imputed with measures from age 43.

*Socioeconomic position*—Own occupational class at age 53 (or if not available, the most recent measure in adulthood (n=74)) was categorised with the registrar general’s social classification into three groups: high (I or II), middle (IIINM or IIIM), or low (IV or V). Educational level attained was recorded at age 26 and categorised into five groups: degree or higher; A levels, usually attained at age 18, or their equivalents; O levels, usually attained at age 16, or their equivalents; CSE, clerical course, or equivalent; none.

*Lifestyle factors*—At age 53 study participants were asked to report whether or not they had participated in any sports, vigorous leisure activities, or exercises in their spare time, not including getting to and from work, in the past four weeks and, if so, on how many occasions they had done these activities. A variable was created that distinguished between those participants who were inactive (reported no participation), moderately active (participated in relevant activities one to four times in the previous four weeks), or most active (participated in relevant activities five or more times in the previous four weeks). The validity of this measure has previously been considered.^23^ Smoking status at 53 was categorised as current, former, or never smoker.

*Health status*—We identified any participants who at age 53 reported a diagnosis of cancer in the previous 10 years; cardiovascular disease (defined as ever having a heart attack or stroke, aortic stenosis, or valvular disease in the past 10 years, angina or Rose angina grade I or II diagnosed by a doctor, or intermittent claudication); and diabetes at any time up to and including age 53. Respiratory symptoms at age 53 were assessed with the UK Medical Research Council’s standardised questions.^28^ We identified a group with the most severe symptoms, who reported one or more of a wheezy or whistling chest most days or nights; usually bringing up phlegm or coughing in the morning or during the day or night in winter for at least three months each year; or more than one chest illness in the past three years that kept them off work or indoors for a week or more.

### Statistical methods

We used Cox proportional hazards regression models to test the associations of each of the three individual measures of physical capability, the composite physical capability score, and the number of tests participants were unable to perform at age 53 with subsequent hazards of all cause mortality. Follow-up time was from March 1999 until confirmed death from any cause, emigration, or the end of March 2012, whichever occurred first. In all initial models the proportional hazards assumption was checked by visual inspection of plots and tests of the Schoenfeld residuals; we found no evidence that the assumption had been violated.

Each of the three individual capability measures and the composite score were included in models as sex specific fifths. For the three individual capability measures, those participants unable to perform a specific test for health reasons were included as a sixth category. This categorisation was used to facilitate the inclusion in analyses of those participants unable to perform the tests for health reasons and to allow for potential deviations from linearity. The upper fifth group was used as reference category in all analyses. To further aid comparisons of associations, we performed all the main analyses on the sample with complete data on all three physical capability measures (that is, those who had a valid score or were coded as unable for health reasons for all three tests) who were also flagged for subsequent mortality follow-up (n=2766).

Initial models were adjusted for sex only, with sex interactions formally tested. Subsequent models were additionally adjusted for body size, socioeconomic position, lifestyle factors, and health status in stages with a final model adjusted for all covariates. To maintain statistical power and minimise the level of potential bias introduced because of missing information, we imputed missing values of the covariates in the sample of 2766 with complete data on physical capability and mortality (BMI (n=29), height (n=5), occupational class (n=24), educational level (n=152), leisure time physical activity (n=2), cancer (n=4), and severe respiratory symptoms (n=3)) using the multiple imputation chained equations implemented in Stata version 12, with inclusion in the imputation model of the event indicator (death) and the Nelson-Aalen estimator of cumulative (baseline) hazard, *H(T)*.^29^ ^30^ All survival analyses described above were hence run across 20 multiply imputed datasets and estimates combined with Rubin’s rules.

To assess the added value of each of the different measures we first investigated the benefit of including multiple measures of physical capability in the same model by running a sex adjusted model with all three measures included. We then used likelihood ratio tests to compare this model with sex adjusted models, including each possible combination of two of the measures. These comparisons assessed the improvement in model fit of including the third measure. To assess the predictive ability of each measure of physical capability and the benefit of including multiple measures in the same model we calculated Harrell’s C index (which estimates the probability of concordance between observed and predicted responses)^31^ for models including each of the three measures separately; the composite score; and all three measures. For the purposes of these calculations, we divided the main analytical sample randomly into derivation and validation sets of equal size, with stratification by sex to ensure the same ratio of men and women in both sets. Sex adjusted models were run in the derivation set and C indices and 95% confidence intervals were then estimated in the validation set.^32^

All analyses were conducted with Stata 12.0 (StataCorp, TX).

### Sensitivity analyses

We ran sensitivity analyses in which we excluded all deaths occurring during the first two years of follow-up (n=12); excluded those participants who had reported any of the specified health status indicators at baseline (n=722); included only those people with complete data on all covariates (that is, excluding those people with imputed data on covariates); and ran the basic sex adjusted models on the maximum available samples for each physical capability test.

## Results

In the main analysis sample (n=2766), 177 deaths (88 from cancer, 47 from cardiovascular disease, and 42 from other causes) occurred between ages 53 and 66 (median and maximum follow-up time 13 years; 5.1 deaths per 1000 person years, 95% confidence interval 4.4 to 5.9). Those participants with lower composite physical capability scores at age 53 had lower socioeconomic position, less healthy lifestyles, and higher prevalence of self reported cardiovascular disease, diabetes, and severe respiratory symptoms compared with those with higher scores (tables 1 and 2 [Table tbl2]).

**Table 1 tbl1:** Characteristics of men at age 53 (271 in each fifth; maximum n=1355) in MRC National Survey of Health and Development stratified by fifths of composite physical capability score (sample restricted to those with data on all three physical capability measures, after inclusion of those unable to perform each test for health reasons). Figures are means (SD) unless stated otherwise

	No*	Total	Fifth of composite physical capability score				
			1 (lowest)	2	3	4	5 (highest)
Grip strength (kg)	1327	47.8 (12.2)	35.7 (12.1)	44.1 (9.28)	49.8 (8.8)	53.9 (9.0)	54.5 (10.9)
Chair rise speed (stands/min)	1291	32.2 (10.4)	22.6 (5.8)	27.5 (6.2)	32.4 (7.7)	36.8 (10.5)	39.3 (10.9)
Standing balance time (s)†	1315	5 (3-10)	3 (2-5)	4 (3-5)	4 (3-6)	7 (4-10)	19 (12-28)
Height (cm)	1351	174.7 (6.6)	173.9 (7.1)	175.1 (6.7)	175.2 (6.6)	174.9 (6.1)	174.2 (6.4)
BMI (kg/m^2^)	1344	27.4 (4.0)	27.3 (4.5)	27.8 (4.0)	27.8 (4.0)	27.4 (4.2)	26.8 (3.5)
Cancer‡	1353	1.6	2.6	1.9	0.7	1.9	0.7
Cardiovascular disease‡	1355	6.3	11.8	7.0	4.1	4.8	3.7
Diabetes‡	1355	3.1	5.5	2.2	2.2	3.3	2.2
Severe respiratory symptoms‡	1353	18.9	22.2	24.0	15.1	17.0	16.2
Occupational class (low-IV or V)¶	1343	11.0	17.2	11.1	13.7	7.4	5.5
Educational level attained (none)¶	1283	35.9	45.7	39.8	36.7	31.6	25.4
Smoking status (current smoker)¶	1355	23.2	27.7	26.2	22.9	22.9	16.2
Leisure time activity level (inactive)¶	1353	47.4	71.5	46.3	49.5	38.4	31.4

*Number varies because of inability of some participants to complete each physical capability test for health reasons (28 (2.1%) for grip strength; 64 (4.7%) for chair rise speed; 40 (3.0%) for standing balance) and missing data on covariates.

†Median (interquartile range).

‡Health status indicators coded as binary variables and figures shown are proportions (%) of men in each category who reported specified condition (see definitions in methods).

¶For brevity, proportion (%) of men in the “highest risk” category of each categorical covariate are presented. Categorisations were: occupational class (high (I or II), middle (IIINM or IIIM), low (IV or V); educational level attained (degree or higher; A levels, usually attained at age 18, or their equivalents; O levels, usually attained at age 16, or their equivalents; CSE, clerical course or equivalent; none); smoking status (current, former, never smoker); leisure time activity level (inactive (no reported activity); moderate (1-4 times a week); high (≥5 times a week)).

**Table 2 tbl2:** Characteristics of women at age 53 (maximum n=1411) in MRC National Survey of Health and Development stratified by fifths of composite physical capability score (sample restricted to those with data on all three physical capability measures, after inclusion of those unable to perform each test for health reasons). Figures are means (SD) unless stated otherwise

	No *	Total	Fifth of composite physical capability score				
			1 (lowest) (n=282)	2 (n=283)	3 (n=283)	4 (n=284)	5 (highest) (n=279)
Grip strength (kg)	1376	27.9 (7.9)	20.6 (6.6)	24.5 (5.3)	27.8 (5.1)	31.7 (6.4)	33.9 (7.9)
Chair rise speed (stands/min)	1339	30.3 (9.3)	21.6 (5.9)	26.6 (5.8)	30.4 (7.1)	33.8 (8.4)	36.8 (10.1)
Standing balance time (s)†	1352	4 (3-7)	3 (2-4)	3 (2-5)	4 (3-6)	5 (3-7)	10 (6-18)
Height (cm)	1410	161.6 (5.9)	160.7 (6.1)	161.6 (6.1)	161.1 (5.9)	162.5 (6.0)	162.2 (5.5)
BMI (kg/m^2^)	1393	27.5 (5.5)	29.6 (6.7)	27.8 (5.3)	27.9 (5.3)	26.5 (4.9)	25.6 (3.9)
Cancer‡	1409	4.3	4.3	4.3	2.5	6.0	4.7
Cardiovascular disease‡	1411	4.8	12.8	5.7	1.1	2.5	2.2
Diabetes‡	1411	2.6	6.4	1.4	2.5	1.8	1.1
Severe respiratory symptoms‡	1410	17.9	25.6	17.7	18.7	14.8	12.9
Occupational class (low-IV or V)¶	1399	21.2	30.6	21.6	20.1	17.1	16.9
Educational level attained (none)¶	1331	36.5	48.0	41.2	38.5	28.6	26.0
Smoking status (current smoker)¶	1411	22.0	25.2	21.6	23.7	22.9	16.9
Leisure time activity level (inactive)¶	1411	50.1	67.0	57.6	50.2	38.7	36.9

*Number varies because of inability of some participants to complete each physical capability test for health reasons (35 (2.5%) for grip strength, 72 (5.1%) for chair rise speed, 59 (4.2%) for standing balance) and missing data on covariates.

†Median (interquartile range).

‡Health status indicators coded as binary variables and figures shown are proportions (%) of women in each category who reported specified condition (see definitions in methods).

¶For brevity, proportion (%) of women in “highest risk” category of each categorical covariate are presented. Categorisations were: occupational class (high (I or II), middle (IIINM or IIIM), low (IV or V); educational level attained (degree or higher; A levels, usually attained at age 18, or their equivalents; O levels, usually attained at age 16, or their equivalents; CSE, clerical course or equivalent; none); smoking status (current, former, never smoker); leisure time activity level (inactive (no reported activity); moderate (1-4 times a week); high (≥5 times a week)).

In sex adjusted models, those participants unable to perform a test and those in the lowest performing fifth of any one of the measures of physical capability had higher mortality rates than those people in the highest fifth (table 3 and figure[Fig fig1]). There was also some evidence of slightly higher rates in other fifths compared with the highest fifth, but there were no clear and consistent patterns of graded association, except for the composite score. Tests of sex interaction suggested some evidence of a stronger association between standing balance time and mortality in men than in women (likelihood ratio test of sex interaction P=0.02), but in both sexes associations were in the same direction and so to maximise power we included men and women together in subsequent models. There was no other evidence of sex interaction. Adjustment for covariates partially attenuated associations, with individual adjustment for lifestyle factors and indicators of health status having the greatest influence (results available on request). In fully adjusted models, however, the finding of higher mortality rates among those unable to perform the tests and in the lowest performing fifth remained (table 3[t3]).

**Figure fig1:**
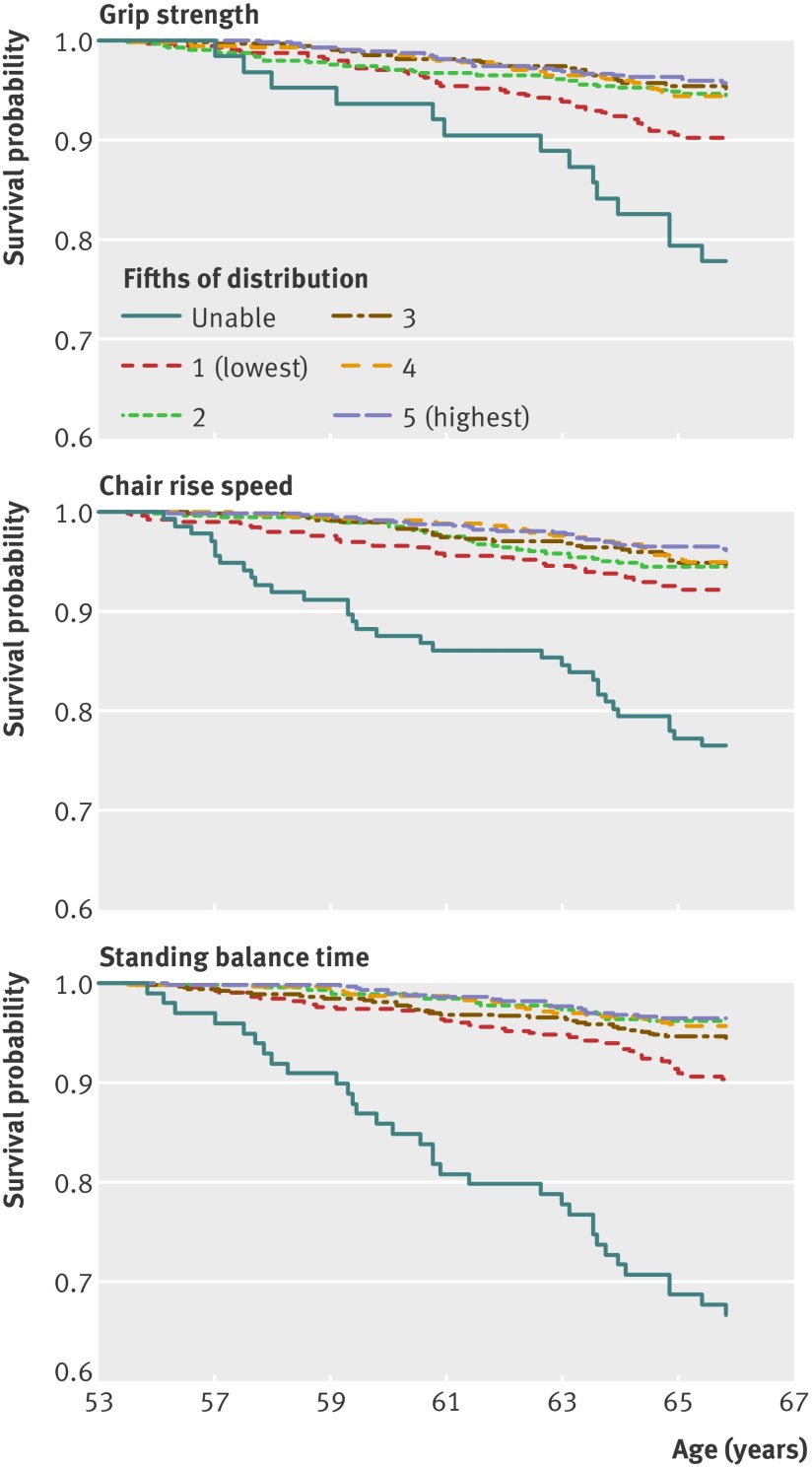
Kaplan Meier survival probabilities stratified by categories of grip strength, chair rise speed, and standing balance time in MRC National Survey of Health and Development (n=2766, 177 deaths)

**Table 3 tbl3:** Associations between performance in objective tests of physical capability at age 53* and rates of death from all causes up to age 66 in MRC National Survey of Health and Development (n=2766, deaths=177). Results are combined from analyses run across 20 imputed datasets

	No in group	No of deaths	Mortality rate per 1000 person years (95% CI)	Hazards ratios for all cause mortality (95% CI) adjusted for:		
				Sex	Sex, height, BMI	All covariates*
Fifths† of grip strength:						
Unable to do test	63	14	18.63 (11.03 to 31.46)	5.84 (3.01 to 11.36)	5.56 (2.83 to 10.89)	4.80 (2.42 to 9.50)
1 (lowest)	540	54	8.01 (6.14 to 10.46)	2.42 (1.49 to 3.94)	2.36 (1.44 to 3.88)	1.98 (1.20 to 3.27)
2	543	30	4.37 (3.06 to 6.26)	1.32 (0.76 to 2.27)	1.31 (0.76 to 2.27)	1.27 (0.73 to 2.21)
3	540	26	3.77 (2.57 to 5.54)	1.13 (0.65 to 1.99)	1.12 (0.64 to 1.97)	1.10 (0.63 to 1.95)
4	539	30	4.37 (3.06 to 6.26)	1.32 (0.76 to 2.27)	1.31 (0.76 to 2.26)	1.29 (0.75 to 2.22)
5 (highest)	541	23	3.33 (3.06 to 6.26)	1.00	1.00	1.00
Fifths† of chair rise speed:						
Unable to do test	136	32	20.47 (14.47 to 28.94)	6.91 (4.02 to 11.90)	6.58 (3.78 to 11.44)	4.28 (2.41 to 7.60)
1 (lowest)	497	40	6.43 (4.72 to 8.77)	2.13 (1.27 to 3.59)	2.12 (1.26 to 3.57)	1.61 (0.94 to 2.76)
2	524	29	4.36 (3.03 to 6.28)	1.44 (0.83 to 2.51)	1.45 (0.83 to 2.53)	1.30 (0.74 to 2.28)
3	470	25	4.17 (2.82 to 6.18)	1.36 (0.77 to 2.41)	1.36 (0.77 to 2.42)	1.21 (0.68 to 2.16)
4	575	29	3.94 (2.74 to 5.67)	1.30 (0.75 to 2.26)	1.30 (0.74 to 2.26)	1.25 (0.72 to 2.19)
5 (highest)	564	22	3.04 (2.00 to 4.62)	1.00	1.00	1.00
Fifths† of standing balance time:						
Unable to do test	99	33	30.28 (21.53 to 42.59)	12.53 (7.16 to 21.91)	11.98 (6.78 to 21.19)	9.61 (5.32 to 17.39)
1 (lowest)	499	49	7.84 (5.92 to 10.37)	3.11 (1.84 to 5.25)	3.05 (1.80 to 5.16)	2.53 (1.48 to 4.33)
2	445	17	2.99 (1.86 to 4.81)	1.17 (0.61 to 2.23)	1.15 (0.60 to 2.20)	1.03 (0.54 to 1.98)
3	632	35	4.37 (3.14 to 6.09)	1.66 (0.96 to 2.87)	1.64 (0.95 to 2.85)	1.65 (0.95 to 2.88)
4	532	23	3.38 (2.25 to 5.09)	1.29 (0.71 to 2.36)	1.29 (0.71 to 2.35)	1.24 (0.68 to 2.27)
5 (highest)	559	20	2.79 (1.80 to 4.33)	1.00	1.00	1.00
Fifths† of composite score:						
1 (lowest)	553	71	10.51 (8.33 to 13.26)	5.38 (3.03 to 9.54)	5.15 (2.89 to 9.17)	3.68 (2.03 to 6.68)
2	554	34	4.84 (3.46 to 6.78)	2.46 (1.32 to 4.58)	2.41 (1.29 to 4.49)	2.17 (1.16 to 4.07)
3	554	31	4.39 (3.09 to 6.24)	2.23 (1.18 to 4.18)	2.18 (1.16 to 4.10)	1.98 (1.05 to 3.74)
4	555	27	3.80 (2.61 to 5.54)	1.92 (1.01 to 3.67)	1.92 (1.00 to 3.66)	1.73 (0.90 to 3.30)
5 (highest)	550	14	1.98 (1.17 to 3.35)	1.00	1.00	1.00
No of tests unable to be performed:						
3	21	11	49.55 (27.44 to 89.47)	12.29 (6.64 to 22.76)	11.62 (6.23 to 21.66)	8.40 (4.35 to 16.23)
2	55	13	20.77 (12.06 to 35.76)	5.56 (3.14 to 9.84)	5.14 (2.86 to 9.26)	3.95 (2.17 to 7.18)
1	125	20	13.45 (8.68 to 20.85)	3.41 (2.13 to 5.46)	3.35 (2.09 to 5.37)	2.81 (1.74 to 4.51)
0	2565	133	4.07 (3.43 to 4.82)	1.00	1.00	1.00

*Sex, body size (height and BMI), socioeconomic position (occupational class and educational level attained), lifestyle factors (smoking status and leisure activity levels), and health status (cancer, cardiovascular disease, diabetes, severe respiratory symptoms).

†For range of values in each sex specific fifth see table D in appendix.

In sex adjusted models, people who could not perform any of the tests had over 12 times the mortality rate compared with the people who were able to perform all three tests (hazard ratio 12.29, 95% confidence interval 6.64 to 22.76). There was a clear graded pattern of association between the number of tests a person was unable to perform and rates of death; and these associations were only partly attenuated by covariates (table 3).

When we included all three measures of physical capability in the same sex adjusted model we still found independent associations between each of the three measures and mortality rates; mutually adjusted hazard ratios for the lowest fifth of performers and those unable to perform compared with the highest fifth were 1.99 (95% confidence interval 1.23 to 3.22), 2.09 (1.27 to 3.41), and 3.40 (2.05 to 5.63) for grip strength, chair rise speed, and standing balance time, respectively. Results from likelihood ratio tests confirmed that inclusion of all three measures improved model fit (see table A in appendix). Inclusion of any one of the measures increased the predictive ability of the model, when compared with a model including only sex, as indicated by an increase in the C index (table 4). A model including all three measures produced the highest (C index (0.71), though a model including only standing balance produced a similar estimate (C index=0.70).

**Table 4 tbl4:** Estimates of Harrell’s C index from models of associations between different combinations of physical capability measures at age 53 and time to death over 13 years of follow-up in MRC National Survey of Health and Development

Variables included in model	C index (95% CI)
Sex	0.54 (0.48 to 0.59)
Grip strength (GS) + sex	0.63 (0.56 to 0.69)
Chair rise speed (CS) + sex	0.66 (0.60 to 0.72)
Standing balance time (SB) + sex	0.70 (0.64 to 0.76)
GS + CS + SB + sex	0.71 (0.65 to 0.77)
Composite score + sex	0.69 (0.63 to 0.74)

Each individual physical capability measure and composite score was included as categorical variables; categorisations as shown in table 3.

### Sensitivity analyses

The main findings were unchanged after we excluded all deaths occurring during the first two years of follow-up and those participants who had at least one of the specified health status indicators (that is, cardiovascular disease, cancer, diabetes, or severe respiratory symptoms) at baseline (see table B in appendix). Adjustments for covariates had similar influences on effect estimates when we ran the main analyses on the sample with complete data on all covariates compared with the influence of covariate adjustments in the main analyses run across 20 imputed datasets. Findings were also similar when we ran analyses on maximum available samples.

## Discussion

We have found robust evidence of associations between three commonly used objective measures of physical capability assessed in mid-life and rates of all cause mortality over 13 years of follow-up. Our findings show that two groups of people consistently experience higher rates of mortality than those in the upper fifth of performance: those who were unable to perform the test and those in the bottom fifth of performance. There was some evidence that all three measures provided added value, as assessed by model fit.

### Comparison with other studies

Since the publication of a previous systematic review,^1^ the association between walking speed and mortality has been shown in the Whitehall II study, which had a slightly younger age at baseline assessment than previous studies (mean age 61),^33^ and an additional study of the association between muscle strength and mortality in a younger population with follow-up over 24 years^34^ has also been published. To the best of our knowledge, however, this is the first study to examine the associations between chair rise speed and standing balance time in mid-life and mortality.

Among those able to perform a specific test, the clearest evidence of associations was found when we compared the lowest and highest fifths, with little clear evidence of differences between the highest and other fifths. This is consistent with recent findings of non-linear associations between strength at ages 16-19 and subsequent mortality in over a million Swedish men.^34^

### Explanation of findings

Inclusion of each one of the three measures of physical capability improved model fit, and the only clear graded pattern of association across all categories of performance that we found was in models of the composite physical capability score, suggesting that when relations with mortality are examined, there is likely to be value in assessing a range of different measures of physical capability in mid-life. This suggests that despite being dependent, at least to some extent, on the same underlying physiological systems, the three measures of physical capability in mid-life are all assessing sufficiently distinct underlying characteristics. The relatively low correlations between the three different measures at age 53 (all values of r<0.25) and the fact that only 40 participants were in the bottom performing fifth of all three measures and only 37 were in the top performing fifth supports this. In some studies of older populations, when multiple measures of physical capability have been mutually adjusted for, not all associations with mortality have been retained,^35^ ^36^ suggesting that the added value of assessing multiple measures might decline with age. Shared risk factors might become more common with increasing age, such as chronic diseases, dysregulation of homeostasis, and concurrent declines in the function of multiple physiological systems.

Our estimates of predictive ability should be interpreted with caution as the C index cannot detect small differences in discriminatory ability between two models.^31^ Furthermore, it has been suggested that it is inappropriate to rely solely on these estimates to evaluate the utility of potential risk predictors, which is why we also considered model fit.^37^ While inclusion of all three measures does improve model fit, standing balance time at age 53 was more strongly related to mortality than either of the two other measures of physical capability. This might to some extent be driven by the finding of stronger associations between inability to perform this test and mortality than other tests. At age 53 details of the specific health problems that prevented participants from being able to perform each test were not recorded. In a more recent assessment of participants (at age 60-64) in the MRC National Survey of Health and Development, however, details of health problems that limited people’s ability to complete the same tests were recorded. At this later age, diseases of the musculoskeletal and cardiovascular systems and injuries were commonly reported reasons for not being able to perform all three tests. The range of reasons provided, however, was more diverse for the standing balance and chair rising tests than grip strength, with respiratory diseases limiting some participant’s ability to perform the chair rising test and symptoms such as dizziness and unsteadiness affecting ability to perform the balance test.

As performance in the standing balance test relies more on neurocognitive function than either of the other two tests,^38^ underlying neurodevelopmental pathways could partially explain our findings. In addition, tests of the association between each measure of physical capability and cause specific mortality (results not shown) showed that standing balance performance was the only one of the three measures associated with deaths from cancer (which was responsible for 50% of deaths), whereas patterns of association between all three measures and deaths from cardiovascular disease and other causes were similar to those presented for all cause mortality. Further studies, with greater statistical power to investigate cause specific death, are required to elucidate the reason for this.

The presence of a stronger association between standing balance time and mortality probably explains why inclusion of all three measures in the same model produced a slightly higher estimate of the C index than inclusion of the composite score. By using a composite score greater restrictions are placed on the model. Each of the three measures is given equal weight and similar strengths of association are assumed; this might not be fully appropriate in this sample at this age.

One often cited explanation for finding associations between objective measures of physical capability and mortality is that they act as general markers of disease, disability, general health status, and ageing. Because of the relatively young age of participants in this study, we consider that the associations found are unlikely to have been driven entirely by disease and ageing processes that are already clinically manifest; less than 4% of study participants had two or more key comorbidities and most (about 73%) had none, which suggests a low disease burden especially compared with older populations.^39^ Furthermore, associations were not restricted to the groups unable to perform the tests and were retained when we adjusted for key indicators of health status and excluded those people who died within two years of follow-up and those people with poor health status at baseline. It is possible, however, that our results could be explained by the influence of subclinical disease and ageing processes on physical capability, with relatively low levels of performance, even in mid-life, providing a useful indication of these processes, despite them not yet being clinically manifest.

Objective measures of physical capability could also be associated with mortality because they reflect lifetime exposure to lifestyle factors related to risk of premature mortality such as physical inactivity, smoking, and unhealthy diet. Adjustment for smoking and leisure time physical activity at baseline did partially attenuate the main associations, providing some evidence in support of this. No further attenuation was seen when we adjusted for a variable indicating cumulative exposure to leisure time physical activity across adulthood (see table C in appendix), suggesting that it is unlikely to fully explain the main associations found.

### Methodological considerations

A key strength of these analyses is the inclusion of three different commonly used objective measures of physical capability, which have been assessed with standardised protocols in mid-life with follow-up of more than 10 years. There was no measure of walking speed, which is considered by some researchers to have more value than other measures of physical capability in older populations.^2^ ^40^ There is no evidence in the literature, however, to suggest that the association between walking speed in mid-life and mortality would be any stronger than the associations we found with other measures. Indeed, the association might be weaker because it has been suggested that at younger ages walking speed has less discriminatory ability than the measures of physical capability examined in this study.^41^

Our study population was homogeneous for age, which enabled us to examine associations free from the strong confounding effect of chronological age; in samples heterogeneous for age, age is consistently found to be one of the strongest predictors of survival and once it is included in the model, inclusion of other factors does little to improve prediction.^42^

Another important strength of the analyses was the inclusion of those people unable to perform each test for health reasons. As different exclusion criteria are applied to different tests of physical capability, and a greater proportion of participants are unable to perform some tests than others, exclusion of these people not only potentially introduces bias and limits the possibility of examining physical capability across the full spectrum of ability but also makes comparisons of the different measures unfair.

Limitations of our analyses include the fact that we had insufficient statistical power to fully investigate cause specific mortality. Some other studies have examined associations between objective measures of physical capability, most often walking speed, and cause specific mortality, with deaths from cardiovascular disease most consistently related, which our findings also suggest.^33^ ^43^ ^44^ An additional limitation of our study is that despite having prospectively collected data on a wide range of potential confounding factors, we are unable to fully exclude the possibility that our results could be explained by residual confounding. This is because of a lack of available data on some potentially important factors, such as fitness; the availability of only limited measures of others (for example, physical activity was self reported and captured only leisure time activity); and our decision a priori not to include other measures, such as nutritional intake and alcohol consumption.

Another limitation is that we could examine physical capability at only one time point. Other recent studies provide evidence to suggest that greater rates of decline in physical capability over time in later life are associated with higher rates of death.^45^ ^46^ ^47^ ^48^ Work is now needed to establish the added value of assessing change over time compared with assessment at just one time point and of using different combinations of data on baseline and change for different combinations of physical capability measures.

The MRC National Survey of Health and Development was established by using a sampling frame that ensured that it was nationally representative of the population born in England, Scotland, and Wales in 1946. Since then there have been losses to follow-up from death, emigration, loss of contact, and permanent refusal. Despite this, at age 53, when we assessed physical capability, in most respects the sample remained representative of the national population born at a similar time.^9^ ^49^ As deaths were ascertained via linkage to a national register and, in these analyses, multiple imputation was used for missing data on covariates, additional losses to follow-up and sample restrictions were limited, which should have minimised bias.

### Implications

Our findings suggest that people with relatively poor performance in mid-life in any one of these tests of physical capability are an important group for intervention, with those people unable to perform one or more of the tests or with poor performance on multiple tests particularly important target groups. Further research is needed to identify the most effective interventions to improve levels of physical capability in mid-life and to test whether these interventions have a positive impact on survival rates. So far, such studies, including one currently underway in the United States, which is examining the impact of a physical activity intervention, have focused on high risk older people.^50^

As inability to perform any one of the tests for health reasons is associated with higher rates of mortality, the exclusion of these groups from analyses is likely to introduce bias. Not all studies that have assessed physical capability have captured data on reasons for missing values.^5^ Our findings highlight the importance of ensuring that the reason for non-completion is captured in sufficient detail so that those people unable to do the tests for health reasons can be identified and handled appropriately in analyses.

Our finding of increased rates of death in the bottom fifth of performance suggests that there might be threshold effects on which to base clinically meaningful cut points. While these measures are not currently in widespread use in primary care, researchers and health professionals have shown growing interest in the possibility of using simple objective measures of physical capability in applied settings, especially in the assessment of older people.^19^ ^51^ Caution is required as strong associations at the population level do not necessarily equate with reliable clinical prediction within individuals.^52^ ^53^ Furthermore, at present absolute cut points identified might vary across studies and those in one study might not be applicable to another as a result of differences in study population characteristics and assessment protocol,^5^ ^19^ with standing balance considered to be the most problematic of the measures as it is assessed in a wide range of different ways in different studies.^1^ ^11^ ^54^ Therefore, to identify absolute cut points that are meaningful and can be applied widely in clinical and non-clinical settings, further work is required to first establish reference data for national populations across the full adult age range with standardised protocols for assessment, which recent initiatives have been developing.^55^

### Conclusions

Our study shows robust associations of standing balance time, chair rise speed, and grip strength at age 53 with all cause mortality rates over 13 years of follow-up. This suggests that there is value in using these simple and inexpensive tests to assess physical capability in mid-life in research and possibly also in applied settings to identify those people (that is, those with relatively low performance and those unable to perform the tests) who are less likely than others to achieve a long and healthy life.

**What is already known on this topic**

Better performance in objective tests of physical capability, including stronger grip and faster walking speed, has consistently been shown to be associated with lower all cause mortality rates in older people living in the community
Though these observed associations might be explained by the pathways between disease pathology, physical capability, and mortality that are operating in later life, it is unclear whether associations are also consistently found in younger populations before these specific pathways are manifest
A systematic review found that there are few existing studies that have assessed the value of conducting more than one different test of physical capability

**What this study adds**

There are robust associations between standing balance time, chair rise speed, and grip strength at age 53 and all cause mortality rates over 13 years of follow-up
Relatively low levels of performance in tests of physical capability in mid-life could provide a useful indication of the presence of underlying disease and ageing processes that are likely to be detrimental for future health but are not yet necessarily clinically manifest
Objective assessment of physical capability in mid-life is of value

## Notes

Contributors: All authors contributed to the conception and design of the study; RC and BHS undertook statistical analyses; RC drafted the article and all authors contributed to its critical revision and provided final approval of the version to be published. RC is guarantor. All authors, external and internal, had full access to all of the data (including statistical reports and tables) in the study and can take responsibility for the integrity of the data and the accuracy of the data analysis.

Ethical approval: Relevant ethical approval was received for this study, with the assessment at 53 approved by the North Thames Multi-Centre research ethics committee. All participants gave informed consent.

Open access: This is an Open Access article distributed in accordance with the terms of the Creative Commons Attribution (CC BY 3.0) license, which permits others to distribute, remix, adapt and build upon this work, for commercial use, provided the original work is properly cited. See: http://creativecommons.org/licenses/by/3.0.

Transparency declaration: RC affirms that the manuscript is an honest, accurate, and transparent account of the study being reported and that no important aspects of the study have been omitted.

Data sharing statement: Bona fide researchers can apply to access the NSHD data via a standard application procedure (further details available at www.nshd.mrc.ac.uk/data.aspx).

## Notes

Cite this as: *BMJ* 2014;348:g2219

## References

[1] Cooper R, Kuh D, Hardy R; Mortality Review Group. Objectively measured physical capability levels and mortality: systematic review and meta-analysis. BMJ 2010;341:c4467. 20829298 10.1136/bmj.c4467 PMC2938886

[2] Studenski S, Perera S, Patel K, Rosano C, Faulkner K, Inzitari M, et al. Gait speed and survival in older adults. JAMA 2011;305:50-8. 21205966 10.1001/jama.2010.1923 PMC3080184

[3] Martin-Ruiz C, Jagger C, Kingston A, Collerton J, Catt M, Davies K, et al. Assessment of a large panel of candidate biomarkers of ageing in the Newcastle 85+ study. Mech Ageing Dev 2011;132:496-502. 21864562 10.1016/j.mad.2011.08.001

[4] Rosero-Bixby L, Dow WH. Predicting mortality with biomarkers: a population-based prospective cohort study for elderly Costa Ricans. Popul Health Metr 2012;10:11. 22694922 10.1186/1478-7954-10-11 PMC3507767

[5] Cooper R, Hardy R, Aihie Sayer A, Ben-Shlomo Y, Birnie K, Cooper C, et al. Age and gender differences in physical capability levels from mid-life onwards: the harmonisation and meta-analysis of data from eight UK cohort studies. PLoS One 2011;6:e27899. 22114723 10.1371/journal.pone.0027899 PMC3218057

[6] Cesari M, Kritchevsky SB, Newman AB, Simonsick EM, Harris TB, Penninx BW, et al. Added value of physical performance measures in predicting adverse health-related events: results from the Health, Aging And Body Composition Study. J Am Geriatr Soc 2009;57:251-9. 19207142 10.1111/j.1532-5415.2008.02126.x PMC2695653

[7] Freiberger E, de Vreede P, Schoene D, Rydwik E, Mueller V, Frandin K, et al. Performance-based physical function in older community-dwelling persons: a systematic review of instruments. Age Ageing 2012;41:712-21. 22885845 10.1093/ageing/afs099

[8] Guralnik JM, Simonsick EM, Ferrucci L, Glynn RJ, Berkman LF, Blazer DG, et al. A short physical performance battery assessing lower extremity function: association with self-reported disability and prediction of mortality and nursing home admission. J Gerontol 1994;49:M85-M94. 8126356 10.1093/geronj/49.2.m85

[9] Wadsworth M, Kuh D, Richards M, Hardy R. Cohort profile: the 1946 national birth cohort (MRC National Survey of Health and Development). Int J Epidemiol 2006;35:49-54. 16204333 10.1093/ije/dyi201

[10] Kuh D, Bassey EJ, Butterworth S, Hardy R, Wadsworth MEJ. Grip strength, postural control, and functional leg power in a representative cohort of British men and women: associations with physical activity, health status, and socioeconomic conditions. J Gerontol Med Sci 2005;60A:224-31. 10.1093/gerona/60.2.224 15814867

[11] Kuh D, Karunananthan S, Bergman H, Cooper R. A life-course approach to healthy ageing: maintaining physical capability. Proc Nutr Soc 2014;1-12. 10.1017/S0029665113003923 PMC3981474 24456831

[12] Kuh D, Hardy R, Butterworth S, Okell L, Wadsworth M, Cooper C, et al. Developmental origins of midlife grip strength: findings from a birth cohort study. J Gerontol Med Sci 2006;61:702-6. 10.1093/gerona/61.7.702 16870632

[13] Bassey EJ. Longitudinal changes in selected physical capabilities: muscle strength, flexibility and body size. Age Ageing 1998;27(suppl 3):12-6. 10408678 10.1093/ageing/27.suppl_3.12

[14] Stark T, Walker B, Phillips JK, Fejer R, Beck R. Hand-held dynamometry correlation with the gold standard isokinetic dynamometry: a systematic review. PM R 2011;3:472-9. 21570036 10.1016/j.pmrj.2010.10.025

[15] Bohannon RW. Test-retest reliability of the five-repetition sit-to-stand test: a systematic review of the literature involving adults. J Strength Cond Res 2011;25:3205-7. 21904240 10.1519/JSC.0b013e318234e59f

[16] Bohannon RW, Bubela DJ, Magasi SR, Gershon RC. Relative reliability of three objective tests of limb muscle strength. Isokinet Exerc Sci 2011;19:77-81. 10.3233/IES-2011-0400 PMC3856906 24339479

[17] Curb JD, Ceria-Ulep CD, Rodriguez BL, Grove J, Guralnik J, Willcox BJ, et al. Performance-based measures of physical function for high-function populations. J Am Geriatr Soc 2006;54:737-42. 16696737 10.1111/j.1532-5415.2006.00700.x

[18] Yim-Chiplis PK, Talbot LA. Defining and measuring balance in adults. Biol Res Nurs 2000;1:321-31. 11232210 10.1177/109980040000100408

[19] Roberts HC, Denison HJ, Martin HJ, Patel HP, Syddall H, Cooper C, et al. A review of the measurement of grip strength in clinical and epidemiological studies: towards a standardised approach. Age Ageing 2011;40:423-9. 21624928 10.1093/ageing/afr051

[20] Guralnik JM, Butterworth S, Wadsworth MEJ, Kuh D. Childhood socioeconomic status predicts physical functioning a half century later. J Gerontol Med Sci 2006;61:694-701. 10.1093/gerona/61.7.694 16870631

[21] Strand BH, Cooper R, Hardy R, Kuh D, Guralnik J. Lifelong socioeconomic position and physical performance in midlife: results from the British 1946 birth cohort. Eur J Epidemiol 2011;26:475-83. 21416275 10.1007/s10654-011-9562-9 PMC3246593

[22] Strand BH, Mishra G, Kuh D, Guralnik JM, Patel KV. Smoking history and physical performance in midlife: results from the British 1946 birth cohort. J Gerontol Med Sci 2011;66A:142-9. 10.1093/gerona/glq199 PMC3011956 21071620

[23] Cooper R, Mishra GD, Kuh D. Physical activity across adulthood and physical performance in midlife: findings from a British Birth Cohort. Am J Prev Med 2011;41:376-84. 21961464 10.1016/j.amepre.2011.06.035 PMC3185208

[24] Strand BH, Kuh D, Shah I, Guralnik J, Hardy R. Childhood, adolescent and early adult body mass index in relation to adult mortality: results from the British 1946 birth cohort. J Epidemiol Community Health 2012;66:225-32. 20889586 10.1136/jech.2010.110155 PMC3491865

[25] Kuh D, Hardy R, Langenberg C, Richards M, Wadsworth MEJ. Mortality in adults aged 26-54 years related to socioeconomic conditions in childhood and adulthood: postwar birth cohort study. BMJ 2002;325:1076-80. 12424168 10.1136/bmj.325.7372.1076 PMC131184

[26] Lee IM, Skerrett PJ. Physical activity and all-cause mortality: what is the dose-response relation? Med Sci Sports Exerc 2001;33:S459-71. 11427772 10.1097/00005768-200106001-00016

[27] Doll R, Peto R, Boreham J, Sutherland I. Mortality in relation to smoking: 50 years’ observations on male British doctors. BMJ 2004;328:1519. 15213107 10.1136/bmj.38142.554479.AE PMC437139

[28] Medical Research Council. Questionnaire on respiratory symptoms and instructions for interviewers. MRC, 1976.

[29] Royston P. Multiple imputation of missing values: further update of ice, with an emphasis on categorical variables. Stata J 2009;9:466-77.

[30] White IR, Royston P. Imputing missing covariate values for the Cox model. Stat Med 2009;28:1982-98. 19452569 10.1002/sim.3618 PMC2998703

[31] Harrell FE, Jr., Lee KL, Mark DB. Multivariable prognostic models: issues in developing models, evaluating assumptions and adequacy, and measuring and reducing errors. Stat Med 1996;15:361-87. 8668867 10.1002/(SICI)1097-0258(19960229)15:4<361::AID-SIM168>3.0.CO;2-4

[32] Newson RB. Comparing the predictive power of survival models using Harrell’s C or Somers’ D. Stata J 2010;10:339-58.

[33] Elbaz A, Sabia S, Brunner E, Shipley M, Marmot M, Kivimaki M, et al. Association of walking speed in late midlife with mortality: results from the Whitehall II cohort study. Age (Dordr) 2013;35:943-52. 22361996 10.1007/s11357-012-9387-9 PMC3636402

[34] Ortega FB, Silventoinen K, Tynelius P, Rasmussen F. Muscular strength in male adolescents and premature death: cohort study of one million participants. BMJ 2012;345:e7279. 23169869 10.1136/bmj.e7279 PMC3502746

[35] Idland G, Engedal K, Bergland A. Physical performance and 13.5-year mortality in elderly women. Scand J Public Health 2013;41:102-8. 23178925 10.1177/1403494812466460

[36] Seino S, Kim MJ, Yabushita N, Nemoto M, Jung S, Osuka Y, et al. Is a composite score of physical performance measures more useful than usual gait speed alone in assessing functional status? Arch Gerontol Geriatr 2012;55:392-8. 22197063 10.1016/j.archger.2011.11.011

[37] Cook NR. Use and misuse of the receiver operating characteristic curve in risk prediction. Circulation 2007;115:928-35. 17309939 10.1161/CIRCULATIONAHA.106.672402

[38] Kuh D, Cooper R, Hardy R, Guralnik J, Richards M. Lifetime cognitive performance is associated with midlife physical performance in a prospective national birth cohort study. Psychosom Med 2009;71:38-48. 19124616 10.1097/PSY.0b013e31818a1620 PMC2890312

[39] Collerton J, Davies K, Jagger C, Kingston A, Bond J, Eccles MP, et al. Health and disease in 85 year olds: baseline findings from the Newcastle 85+ cohort study. BMJ 2009;339:b4904. 20028777 10.1136/bmj.b4904 PMC2797051

[40] Lusardi MM. Is walking speed a vital sign? Absolutely. Top Geriatr Rehabil 2012;28:67-76.

[41] Melzer D, Gardener E, Lang I, McWilliams B, Guralnik JM. Measured physical performance. In: Banks J, Breeze E, Lessof C, Nazroo J, eds. Retirement, health and relationships of the older population in England: the 2004 English longitudinal study of ageing (wave 2). Institute for Fiscal Studies, 2006.

[42] Walter S, Mackenbach J, Voko Z, Lhachimi S, Ikram MA, Uitterlinden AG, et al. Genetic, physiological, and lifestyle predictors of mortality in the general population. Am J Public Health 2012;102:e3-10. 10.2105/AJPH.2011.300596 PMC3489349 22397355

[43] Dumurgier J, Elbaz A, Ducimetiere P, Tavernier B, Alperovitch A, Tzourio C. Slow walking speed and cardiovascular death in well functioning older adults: prospective cohort study. BMJ 2009;339:b4460. 19903980 10.1136/bmj.b4460 PMC2776130

[44] Von Bonsdorff MB, Groffen DA, Vidal JS, Rantanen T, Jonsson PV, Garcia M, et al. Coronary artery calcium and physical performance as determinants of mortality in older age: the AGES-Reykjavik Study. Int J Cardiol 2013;168:2094-9. 23414742 10.1016/j.ijcard.2013.01.067 PMC3674198

[45] Xue QL, Beamer BA, Chaves PH, Guralnik JM, Fried LP. Heterogeneity in rate of decline in grip, hip, and knee strength and the risk of all-cause mortality: the Women’s Health and Aging Study II. J Am Geriatr Soc 2010;58:2076-84. 21054287 10.1111/j.1532-5415.2010.03154.x PMC3058914

[46] Hirsch CH, Buzkova P, Robbins JA, Patel KV, Newman AB. Predicting late-life disability and death by the rate of decline in physical performance measures. Age Ageing 2012;41:155-61. 22156556 10.1093/ageing/afr151 PMC3280678

[47] Sabia S, Dumurgier J, Tavernier B, Head J, Tzourio C, Elbaz A. Change in fast walking speed preceding death: results from a prospective longitudinal cohort study. J Gerontol A Biol Sci Med Sci 2014;69:354-62. 23913931 10.1093/gerona/glt114 PMC3976141

[48] White DK, Neogi T, Nevitt MC, Peloquin CE, Zhu Y, Boudreau RM, et al. Trajectories of gait speed predict mortality in well-functioning older adults: the Health, Aging and Body Composition study. J Gerontol A Biol Sci Med Sci 2013;68:456-64. 23051974 10.1093/gerona/gls197 PMC3593620

[49] Wadsworth MEJ, Butterworth SL, Hardy RJ, Kuh DJ, Richards M, Langenberg C, et al. The life course prospective design: an example of benefits and problems associated with longevity. Soc Sci Med 2003;57:2193-205. 14512249 10.1016/s0277-9536(03)00083-2

[50] Fielding RA, Rejeski WJ, Blair S, Church T, Espeland MA, Gill TM, et al. The lifestyle interventions and independence for elders study: design and methods. J Gerontol A Biol Sci Med Sci 2011;66:1226-37. 21825283 10.1093/gerona/glr123 PMC3193523

[51] Garcia-Pena C, Garcia-Fabela LC, Gutierrez-Robledo LM, Garcia-Gonzalez JJ, Arango-Lopera VE, Perez-Zepeda MU. Handgrip strength predicts functional decline at discharge in hospitalized male elderly: a hospital cohort study. PLoS One 2013;8:e69849. 23936113 10.1371/journal.pone.0069849 PMC3723742

[52] Ware JH. The limitations of risk factors as prognostic tools. N Engl J Med 2006;355:2615-7. 17182986 10.1056/NEJMp068249

[53] Grady D, Berkowitz SA. Why is a good clinical prediction rule so hard to find? Arch Intern Med 2011;171:1701-2. 22025427 10.1001/archinternmed.2011.482

[54] Deandrea S, Lucenteforte E, Bravi F, Foschi R, La VC, Negri E. Risk factors for falls in community-dwelling older people: a systematic review and meta-analysis. Epidemiology 2010;21:658-68. 20585256 10.1097/EDE.0b013e3181e89905

[55] Reuben DB, Magasi S, McCreath HE, Bohannon RW, Wang YC, Bubela DJ, et al. Motor assessment using the NIH Toolbox. Neurology 2013;80(11 suppl 3):S65-75. 23479547 10.1212/WNL.0b013e3182872e01 PMC3662336

