# Supplementary material for: Physical capability in mid-life and survival over 13 years of follow-up: British birth cohort study
Source: BMJ. 2014 Apr 29;348:g2219. doi: 10.1136/bmj.g2219 (PMC4004787; doi:10.1136/bmj.g2219)
Supplement: Supplementary file 1 [file coor015836.ww1_default.pdf]

Appendix: Supplementary material [posted as supplied by author]

Supplementary methods

The three physical capability test scores were rescaled to a 0-1 scale as follows. Grip strength was first adjusted for body size by dividing it by height. This measure of height-adjusted grip strength was then divided by the sex-specific value of the 99th percentile (0.4346 kg/cm for men and 0.2838 kg/cm for women), with those participants with values greater than these assigned these values. Chair rise time was rescaled using the equation 1 - (time/44.0 s), where 44.0 was the 99th percentile of time. Participants taking longer than 44.0 seconds were assigned this time. Standing balance time was rescaled by dividing the total time the stand with eyes closed was held by the maximum possible time (i.e. 30 seconds).

Supplementary results

Table A: Results from likelihood ratio tests comparing models with the inclusion of different combinations of physical capability measures (N=2766 (deaths=177))

Models compared		
**Model 1**	**Model 2**	**p-value**
Grip strength (GS) + Sex	Sex	<0.001
Chair rise speed (CS) + Sex	Sex	<0.001
Standing balance time (SB) + Sex	Sex	<0.001
GS + CS + SB + Sex	CS + SB + Sex	0.01
GS + CS + SB + Sex	GS + SB + Sex	0.01
GS + CS + SB + Sex	GS + CS + Sex	<0.001

Each physical capability measure was included as a five category variable: unable or Q1 (lowest performance); Q2; Q3; Q4; Q5 (highest performance)

Table B: Sensitivity analyses – Sex-adjusted associations between different measures of physical capability at age 53 years and rates of death from all causes up to age 66 years in the MRC National Survey of Health and Development with various exclusion criteria applied

	Sex adjusted hazards ratios for all-cause mortality (95% CI)		
**Model*:**	**1**	**2**	**3**
**Grip strength**			
Unable	5.84 (3.01 to 11.36)	5.90 (3.03 to 11.47)	11.43 (5.04 to 25.91)
Q1 (lowest)	2.42 (1.49 to 3.94)	2.29 (1.40 to 3.75)	3.03 (1.51 to 6.07)
2	1.32 (0.76 to 2.27)	1.10 (0.62 to 1.93)	1.09 (0.48 to 2.46)
3	1.13 (0.65 to 1.99)	1.09 (0.62 to 1.92)	1.61 (0.76 to 3.41)
4	1.32 (0.76 to 2.27)	1.19 (0.68 to 2.07)	1.37 (0.63 to 2.99)
Q5 (highest)	1.00	1.00	1.00
**Chair rise speed**			
Unable	6.91 (4.02 to 11.90)	6.63 (3.78 to 11.62)	8.89 (4.25 to 18.62)
Q1 (lowest)	2.13 (1.27 to 3.59)	1.96 (1.14 to 3.36)	2.19 (1.06 to 4.52)
2	1.44 (0.83 to 2.51)	1.41 (0.80 to 2.49)	1.42 (0.66 to 3.08)
3	1.36 (0.77 to 2.41)	1.36 (0.76 to 2.45)	1.56 (0.73 to 3.33)
4	1.30 (0.75 to 2.26)	1.36 (0.78 to 2.38)	1.65 (0.81 to 3.38)
Q5 (highest)	1.00	1.00	1.00
**Standing balance time**			
Unable	12.53 (7.16 to 21.91)	12.22 (6.85 to 21.79)	19.78 (9.02 to 43.36)
Q1 (lowest)	3.11 (1.84 to 5.25)	3.16 (1.85 to 5.41)	3.80 (1.75 to 8.26)
2	1.17 (0.61 to 2.23)	1.16 (0.60 to 2.26)	1.78 (0.74 to 4.32)
3	1.66 (0.96 to 2.87)	1.55 (0.88 to 2.75)	2.48 (1.14 to 5.39)
4	1.29 (0.71 to 2.36)	1.31 (0.71 to 2.42)	1.43 (0.59 to 3.46)
Q5 (highest)	1.00	1.00	1.00
**Composite score**			
Q1 (lowest)	5.38 (3.03 to 9.54)	5.33 (2.94 to 9.66)	8.51 (3.60 to 20.09)
2	2.46 (1.32 to 4.58)	2.41 (1.26 to 4.61)	2.62 (1.01 to 6.82)
3	2.23 (1.18 to 4.18)	2.24 (1.17 to 4.32)	4.12 (1.69 to 10.09)
4	1.92 (1.01 to 3.67)	2.07 (1.07 to 4.02)	2.48 (0.95 to 6.45)
Q5 (highest)	1.00	1.00	1.00
**No. of tests unable**			
3	12.29 (6.64 to 22.76)	13.32 (7.19 to 24.70)	22.66 (11.31 to 45.38)
2	5.56 (3.14 to 9.84)	5.06 (2.73 to 9.39)	6.13 (2.82 to 13.32)
1	3.41 (2.13 to 5.46)	3.29 (2.01 to 5.39)	3.23 (1.62 to 6.47)
0	1.00	1.00	1.00

* Model 1: Sex adjusted model from main analyses (see table 3, presented for comparison with other estimates) (N=2766, 177 deaths)

Model 2: Deaths occurring during first 2 years of follow-up excluded (N=2754, 165 deaths)

Model 3: Participants with any of specified health status indicators (i.e. cancer, CVD, diabetes, severe respiratory symptoms) at baseline excluded (N=2044, 97 deaths)

Table C: Associations between performance in objective tests of physical capability at age 53 years and rates of death from all causes up to age 66 years in the MRC National Survey of Health and Development (N=2766 (deaths=177)) with adjustment for lifestyle factors

	Adjusted hazards ratios for all-cause mortality (95% CI)		
**Model*:**	**1**	**2**	**3**
**Grip strength**			
Unable	5.84 (3.01 to 11.36)	5.00 (2.55 to 9.79)	5.13 (2.62 to 10.04)
Q1 (lowest)	2.42 (1.49 to 3.94)	2.19 (1.34 to 3.58)	2.21 (1.35 to 3.61)
2	1.32 (0.76 to 2.27)	1.27 (0.74 to 2.19)	1.26 (0.73 to 2.18)
3	1.13 (0.65 to 1.99)	1.09 (0.62 to 1.91)	1.06 (0.60 to 1.86)
4	1.32 (0.76 to 2.27)	1.29 (0.75 to 2.22)	1.27 (0.74 to 2.19)
Q5 (highest)	1.00	1.00	1.00
**Chair rise speed**			
Unable	6.91 (4.02 to 11.90)	5.06 (2.89 to 8.83)	5.23 (2.96 to 9.23)
Q1 (lowest)	2.13 (1.27 to 3.59)	1.78 (1.05 to 3.01)	1.78 (1.05 to 3.05)
2	1.44 (0.83 to 2.51)	1.32 (0.76 to 2.30)	1.33 (0.76 to 2.33)
3	1.36 (0.77 to 2.41)	1.23 (0.69 to 2.19)	1.24 (0.70 to 2.22)
4	1.30 (0.75 to 2.26)	1.23 (0.71 to 2.15)	1.22 (0.70 to 2.13)
Q5 (highest)	1.00	1.00	1.00
**Standing balance time**			
Unable	12.53 (7.16 to 21.91)	9.76 (5.51 to 17.26)	10.52 (5.94 to 18.64)
Q1 (lowest)	3.11 (1.84 to 5.25)	2.63 (1.55 to 4.46)	2.70 (1.59 to 4.59)
2	1.17 (0.61 to 2.23)	1.03 (0.54 to 1.97)	1.06 (0.55 to 2.04)
3	1.66 (0.96 to 2.87)	1.54 (0.89 to 2.67)	1.57 (0.90 to 2.73)
4	1.29 (0.71 to 2.36)	1.22 (0.67 to 2.22)	1.28 (0.70 to 2.34)
Q5 (highest)	1.00	1.00	1.00
**Composite score**			
Q1 (lowest)	5.38 (3.03 to 9.54)	4.22 (2.36 to 7.57)	4.39 (2.44 to 7.90)
2	2.46 (1.32 to 4.58)	2.15 (1.15 to 4.03)	2.25 (1.20 to 4.21)
3	2.23 (1.18 to 4.18)	1.94 (1.03 to 3.66)	2.00 (1.06 to 3.77)
4	1.92 (1.01 to 3.67)	1.77 (0.93 to 3.38)	1.81 (0.95 to 3.45)
Q5 (highest)	1.00	1.00	1.00
**No. of tests unable**			
3	12.29 (6.64 to 22.76)	9.69 (5.17 to 18.15)	10.64 (5.65 to 20.02)
2	5.56 (3.14 to 9.84)	4.26 (2.38 to 7.61)	4.43 (2.47 to 7.95)
1	3.41 (2.13 to 5.46)	3.03 (1.89 to 4.87)	3.02 (1.88 to 4.87)
0	1.00	1.00	1.00

* Model 1: Sex adjusted model from main analyses (see table 3, presented for comparison with other estimates)
Model 2: Adjustment made for sex, smoking and physical activity at age 53 (variables modelled as in table 3)
Model 3: Adjustment made for sex, smoking and a cumulative physical activity score

A cumulative physical activity score was derived by assigning those classified as inactive a value of 0, those as moderately active a value of 1 and those as most active a value of 2 at each of the 3 ages across adulthood that leisure time physical activity had been assessed (36, 43 and 53 years) and then summing the values for the 3 ages. The score has values ranging from 0 to 6; a score of 0 indicates that a participant reported being inactive at all 3 ages and a score of 6 indicates that a participant reported being most active at all 3 ages (see Cooper et al, Am J Prev Med 2011;41(4):376-384).

Table D Range of values in each fifth of each physical capability measure by sex

Fifths of measure	Men	Women
Grip strength (kg):		
1	2.8-38.3	2.3-21.1
2	38.4-45.6	21.2-26.1
3	45.7-51.2	26.2-29.7
4	51.3-57.5	29.8-34.1
5	57.6-88.8	34.2-79.5
Chair rise speed (stands/minute):		
1	10.9-23.1	7.5-22.2
2	23.2-27.3	22.3-27.0
3	27.3-31.6	27.1-31.4
4	31.7-37.5	31.5-35.3
5	37.6-120.0	35.4-75.0
Standing balance time (s)*:		
1	0-2	
2	3	
3	4-5	
4	6-9	
5	10-30	
Composite score:		
1	0-1.092	0-1.018
2	1.093-1.290	1.019-1.215
3	1.291-1.460	1.216-1.351
4	1.461-1.667	1.352-1.530
5	1.668-2.795	1.531-2.571

*Same cut-points applied for men and women.
